# Programmed death-ligand 1 regulates ameloblastoma growth and recurrence

**DOI:** 10.1038/s41368-025-00364-w

**Published:** 2025-04-16

**Authors:** Linzhou Zhang, Hao Lin, Jiajie Liang, Xuanhao Liu, Chenxi Zhang, Qiwen Man, Ruifang Li, Yi Zhao, Bing Liu

**Affiliations:** 1 State Key Laboratory of Oral & Maxillofacial Reconstruction and Regeneration, Key Laboratory of Oral Biomedicine Ministry of Education, Hubei Key Laboratory of Stomatology, School & Hospital of Stomatology, Wuhan University, Wuhan, China; 2 Department of Oral and Maxillofacial Surgery, School and Hospital of Stomatology, Wuhan University, Wuhan, China; 3 Department of Prosthodontics, School and Hospital of Stomatology, Wuhan University, Wuhan, China

**Keywords:** Mechanisms of disease, Cell signalling

## Abstract

Tumor cell-intrinsic programmed death-ligand 1 (PD-L1) signals mediate tumor initiation, progression and metastasis, but their effects in ameloblastoma (AM) have not been reported. In this comprehensive study, we observed marked upregulation of PD-L1 in AM tissues and revealed the robust correlation between elevated PD-L1 expression and increased tumor growth and recurrence rates. Notably, we found that PD-L1 overexpression markedly increased self-renewal capacity and promoted tumorigenic processes and invasion in hTERT^+^-AM cells, whereas genetic ablation of PD-L1 exerted opposing inhibitory effects. By performing high-resolution single-cell profiling and thorough immunohistochemical analyses in AM patients, we delineated the intricate cellular landscape and elucidated the mechanisms underlying the aggressive phenotype and unfavorable prognosis of these tumors. Our findings revealed that hTERT^+^-AM cells with upregulated PD-L1 expression exhibit increased proliferative potential and stem-like attributes and undergo partial epithelial‒mesenchymal transition. This phenotypic shift is induced by the activation of the PI3K-AKT-mTOR signaling axis; thus, this study revealed a crucial regulatory mechanism that fuels tumor growth and recurrence. Importantly, targeted inhibition of the PD-L1-PI3K-AKT-mTOR signaling axis significantly suppressed the growth of AM patient-derived tumor organoids, highlighting the potential of PD-L1 blockade as a promising therapeutic approach for AM.

## Introduction

Ameloblastoma (AM) is the most prevalent odontogenic epithelial tumor and is notorious for its aggressive local bone destruction and high recurrence rate.^1,2^ AM, which originates from remnants of odontogenic epithelium, predominantly affects the jaw bones, leading to significant facial deformities and functional impairments. Despite radical surgical interventions, the recurrence rate for conventional AM remains alarmingly high, at approximately 40%–80%.^3–5^ Surgical resection, while necessary, often results in the loss of facial bones, contributing to severe disfigurement and functional challenges. Many AM cases (33%–92%) exhibit the BRAFV600E mutation.^6–8^ Vemurafenib, a targeted inhibitor of the BRAFV600E mutation, has been explored by some clinicians as a treatment option for patients with AM.^8^ However, its efficacy is significantly compromised due to the high incidence of drug resistance. To expand the treatment landscape, further research is imperative to elucidate the underlying mechanisms driving AM pathogenesis. Such insights may pave the way for the development of novel pharmacological therapies to complement existing surgical approaches in the management of AM.

Programmed death-ligand 1 (PD-L1) (B7-H1 or CD274) is a critical immune signaling molecule within the B7 homology (B7-H) family and is recognized primarily for its ability to suppress antitumor T-cell responses by binding PD-1 on T cells.^9,10^ While extensive research has focused on this immune-modulatory function, emerging evidence highlights that PD-L1 exerts significant intrinsic effects within tumor cells, impacting various cellular processes and supporting tumorigenesis beyond immune suppression.^11^ These intrinsic roles of PD-L1 include promotion of tumor initiation, enhancement of metastatic potential, and promoting of tumor progression.^12–14^ PD-L1 has been shown to localize within different cellular compartments (such as the nucleus, cytoplasm, and mitochondria) where it influences diverse cellular functions.^12,13,15,16^ For example, nuclear PD-L1 translocation, which is regulated by pathways involving HDAC2^17^ and p-STAT3,^13^ facilities resistance to anti-PD-1 therapies by modulating the expression of genes related to immune responses and DNA repair mechanisms. Moreover, PD-L1 has been reported to promote homologous recombination repair by interacting with BRCA1, which subsequently affects cell sensitivity to PARP inhibitors, thus contributing to chemoresistance.^18^ Notably, recent findings indicate that more than 50% of AM patients exhibit PD-L1 expression, indicating a role for PD-L1 in AM pathogenesis.^19,20^ However, the specific intrinsic roles of PD-L1 in the growth and recurrence of AM, as well as the underlying mechanisms, remain largely unexplored. Further studies on these aspects may provide valuable insights into AM pathology and reveal new options for targeted therapy.

## Results

### PD-L1 is abnormally overexpressed in ameloblastoma, and this phenotype is correlated with tumor growth and recurrence

**Fig. 1:**
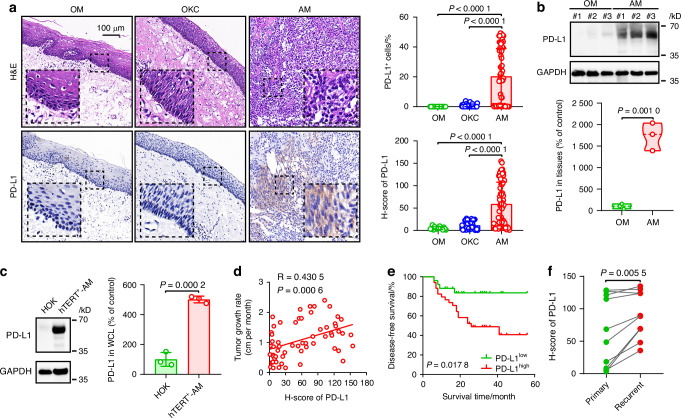
Aberrant overexpression of PD-L1 is observed in ameloblastoma and is correlated with tumor growth and recurrence. **a** Representative hematoxylin and eosin (H&E) and immunohistochemical (IHC) staining of PD-L1 expression in oral mucosa (OM), odontogenic keratocyst (OKC), and ameloblastoma (AM) tissues (left). Data quantification for the histoscore (H-score) is shown on the right, with each point representing one tissue sample. Scale bar, 100 µm. **b** Western blot analysis of PD-L1 expression in OM and AM tissues. **c** Western blot analysis of PD-L1 expression levels in HOK and hTERT^+^-AM cells. **d** Pearson correlation analysis of PD-L1 expression and the tumor growth rate. The tumor growth rate was defined as the largest tumor diameter divided by the duration of symptoms in months (cm/month). **e** Disease-free survival of AM patients with low PD-L1 expression and high PD-L1 expression. **f** Comparison of PD-L1 expression levels between primary and recurrent AM tissues. Mean ± SD, two-tailed unpaired Student’s *t* test (**a**, **b**, **c**) and two-tailed unpaired Student’s *t* test (**f**). Log-rank test (**e**). All results are representative of at least three independent experiments

To assess the expression profile of PD-L1 in ameloblastoma (AM) tissues, we initially employed immunohistochemistry (IHC) to assess PD-L1 expression levels in oral mucosa (OM), odontogenic keratocyst (OKC), and AM tissues. Our results revealed that both the percentage and intensity of PD-L1 expression were significantly greater in AM tissues than in OM and OKC tissues (Fig. 1a). This finding was further corroborated by Western blot analysis, which confirmed elevated PD-L1 levels in AM tissues relative to those in OM tissues (Fig. 1b). Immunofluorescence (IF) analysis revealed that PD-L1 is predominantly expressed in epithelial cells within tumor tissues (Fig. S1a). Additionally, PD-L1 expression was analyzed in human telomerase reverse transcriptase ameloblastoma (hTERT^+^-AM) cell lines and human oral keratinocyte (HOK) cell lines via IF. Confocal microscopy revealed a significantly greater percentage and mean fluorescence intensity (MFI) of PD-L1 expression in hTERT^+^-AM cells than in HOK cells (Fig. S1b). This increase in PD-L1 expression in hTERT^+^-AM cells relative to HOK cells was confirmed by Western blot analysis (Fig. 1c). These results demonstrate that PD-L1 is predominantly and highly expressed in AM epithelial cells.

The aggressive growth and recurrence of tumors are two critical characteristics of AM. Given the abnormal overexpression of PD-L1 in AM, we investigated the correlation between PD-L1 expression and clinical outcomes related to aggressive tumor growth and recurrence. Our analysis revealed a positive correlation between PD-L1 expression levels and the tumor growth rate in AM patients (Fig. 1d). Notably, our findings further demonstrated that AM patients with high PD-L1 expression had a markedly lower disease-free survival rate than did those with low PD-L1 expression (Fig. 1e). Moreover, higher PD-L1 expression was detected in the tissues of recurrent AM patients than in those of primary AM patients (Fig. 1f). Collectively, these findings indicate that elevated PD-L1 expression is associated with enhanced tumor growth and an increased recurrence risk in AM patients.

### PD-L1 regulates the self-renewal capacity, tumorigenesis, and invasiveness of AM cells in vitro

**Fig. 2:**
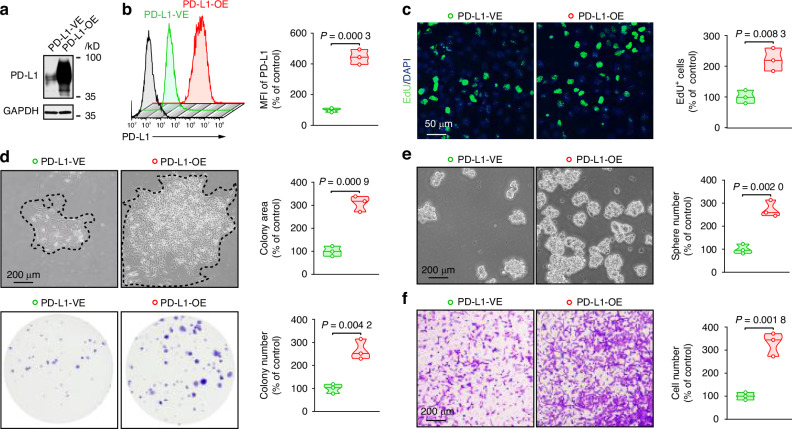
Overexpression of PD-L1 increases the self-renewal capacity, tumorigenicity, and invasiveness of hTERT+-AM cells. **a** Western blot analysis of PD-L1 expression in PD-L1 vector (PD-L1-VE) and PD-L1-overexpressing (PD-L1-OE) hTERT^+^-AM cells. **b** Flow cytometry analysis of PD-L1 expression in PD-L1-VE and PD-L1-OE hTERT^+^-AM cells. **c** Representative images of EdU staining (green) of PD-L1-VE and PD-L1-OE h-TERT^+^-AM cells, with nuclei counterstained with DAPI (blue). Scale bar, 50 µm. **d** Representative images showing the colony formation of PD-L1-VE and PD-L1-OE hTERT^+^-AM cells. Quantitative analysis of the colony area and crystal violet-stained colonies (right). Scale bar, 200 µm. **e** Representative images of spheroid formation in PD-L1-VE and PD-L1-OE hTERT^+^-AM cells (left). Quantitative analysis of the number of spheres (right). Scale bar, 200 µm. **f** Representative images showing the invasive ability of PD-L1-VE and PD-L1-OE hTERT^+^-AM cells (left). Quantitative analysis of the number of invading cells (right). Mean ± SD, two-tailed unpaired Student’s *t* test (**b**–**f**). All the results presented are representative of findings from three independent experiments

After confirming the crucial role of PD-L1 in fostering tumor growth and recurrent behavior in AM patients, we assessed whether PD-L1 modulates cellular activities, including proliferation, self-renewal, and migration, in vitro. To this end, we employed lentiviral vectors to stably overexpress PD-L1 in hTERT^+^-AM cell lines (yielding PD-L1-OE cells). The efficacy of PD-L1 overexpression was confirmed via Western blot and flow cytometry analyses (Fig. 2a, b). Subsequent EdU proliferation assays revealed a substantial increase in the proliferative activity of PD-L1-OE cells compared to that of control cells (Fig. 2c). Moreover, colony formation assays revealed that compared to PD-L1-vector-expressing (PD-L1-VE) cells, PD-L1-OE cells possessed a significantly greater clonogenicity, as evidenced by the formation of larger and more numerous colonies (Fig. 2d). Sphere formation assays further confirmed the enhanced self-renewal capacity of PD-L1-OE cells (Fig. 2e). To assess the invasive phenotype, we conducted wound healing and Matrigel invasion assays. Compared with PD-L1-VE cells, PD-L1-OE cells exhibited notably accelerated wound closure and a significantly greater number of invading cells, indicating increased migratory and invasive properties (Fig. 2f and S2).

To validate the tumor-promoting activity of PD-L1 in hTERT^+^-AM cells, we transfected cells with specific single guide RNAs (sgRNAs) targeting PD-L1 (sgPD-L1) or a control sgRNA (sgCTL) and assessed the differences in cell behaviors. The effect of metformin, a drug known to reduce PD-L1 levels, was also assessed in hTERT^+^-AM cells. The efficacy of PD-L1 inhibition was confirmed via Western blotting (Fig. S3a, b). EdU assays revealed that the number of EdU-positive hTERT^+^-AM cells was significantly lower in the sgPD-L1 transfection group than in the sgCTL transfection group, indicating suppression of proliferation (Fig. S4a). Similarly, metformin treatment led to a marked decrease in the number of EdU-positive cells (Fig. S4b), suggesting that this agent can suppress hTERT^+^-AM cell proliferation. Furthermore, colony formation assays revealed that targeting PD-L1 with sgPD-L1 or metformin significantly reduced the number and size of colonies formed compared with those in the control group (Fig. S4c, d). Sphere formation assays also revealed decreased self-renewal capabilities in sgPD-L1- and metformin-treated hTERT^+^-AM cells (Fig. S4e, f), indicating the suppression of stem-like properties. In invasion assays, sgPD-L1 and metformin treatment significantly inhibited the migratory and invasive properties of hTERT^+^-AM cells (Fig. S4g, h), further confirming that PD-L1 increases cell aggressiveness. In summary, these findings demonstrate that PD-L1 positively regulates hTERT^+^-AM cell activity, thereby promoting tumor growth and recurrence, whereas its inhibition or downregulation through sgPD-L1 transfection or metformin treatment attenuates these aggressive phenotypes.

### Single-cell RNA sequencing revealed that PD-L1 regulates the proliferation, stemness and partial epithelial‒mesenchymal transition of ameloblastoma cells

**Fig. 3:**
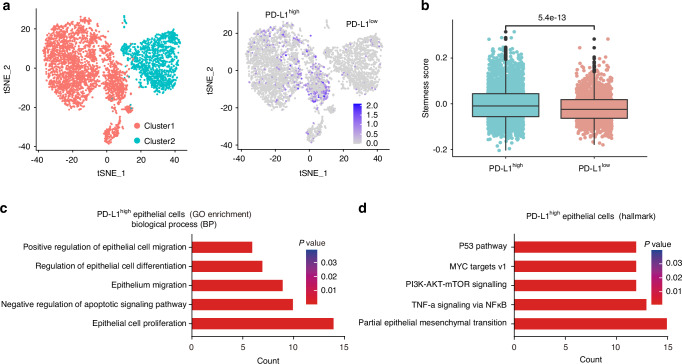
Single-cell RNA sequencing revealed the pivotal role of PD-L1 in regulating cell proliferation, stem-like properties, and partial epithelial-mesenchymal transition characteristics in ameloblastoma. **a** t-SNE plots highlighting different clusters of epithelial cells with PD-L1^Low^ and PD-L1^High^ expression. **b** Boxplot comparing stemness scores in epithelial cells with PD-L1^Low^ and PD-L1^High^ expression. **c** Gene Ontology (GO) analysis of key differentially expressed genes in PD-L1^High^ epithelial cells. **d** Hallmark gene set enrichment analysis of PD-L1^High^ epithelial cells. Mean ± SD, two-tailed unpaired Student’s *t* test (**b**)

To elucidate the mechanism underlying PD-L1-mediated tumor cell aggressive activity in AM, we conducted single-cell RNA sequencing on samples collected from two patients. Four major cell types (epithelial cells, endothelial cells, myeloid cells, and fibroblasts) were identified according to their distinct gene expression profiles (Fig. S5a, b). Further analysis clustered epithelial cells into two major groups according to PD-L1 expression levels (PD-L1^High^ and PD-L1^Low^) (Fig. 3a). Given the significant role of stemness in the recurrence and growth of AM, we compared the stemness scores between the PD-L1^High^ and PD-L1^Low^ groups. Compared with the PD-L1^Low^ group, the PD-L1^High^ group presented significantly higher stemness scores (Fig. 3b), suggesting that PD-L1 plays an important role in maintaining the stemness of AM cells. Gene Ontology (GO) enrichment analysis of the PD-L1^High^ group further revealed that epithelial cells with high PD-L1 levels are involved primarily in biological processes such as cell migration, differentiation, and proliferation, as well as the regulation of apoptotic signaling pathways (Fig. 3c). These processes are related to partial epithelial‒mesenchymal transition (p-EMT) (Fig. 3d). In summary, these findings indicate that PD-L1 plays a crucial role in maintaining stemness and promoting p-EMT, thereby mediating tumor growth and recurrence in AM.

### Proteins related to proliferation, stemness, and partial epithelial-mesenchymal transition are overexpressed in human ameloblastoma tissues and cell lines

**Fig. 4:**
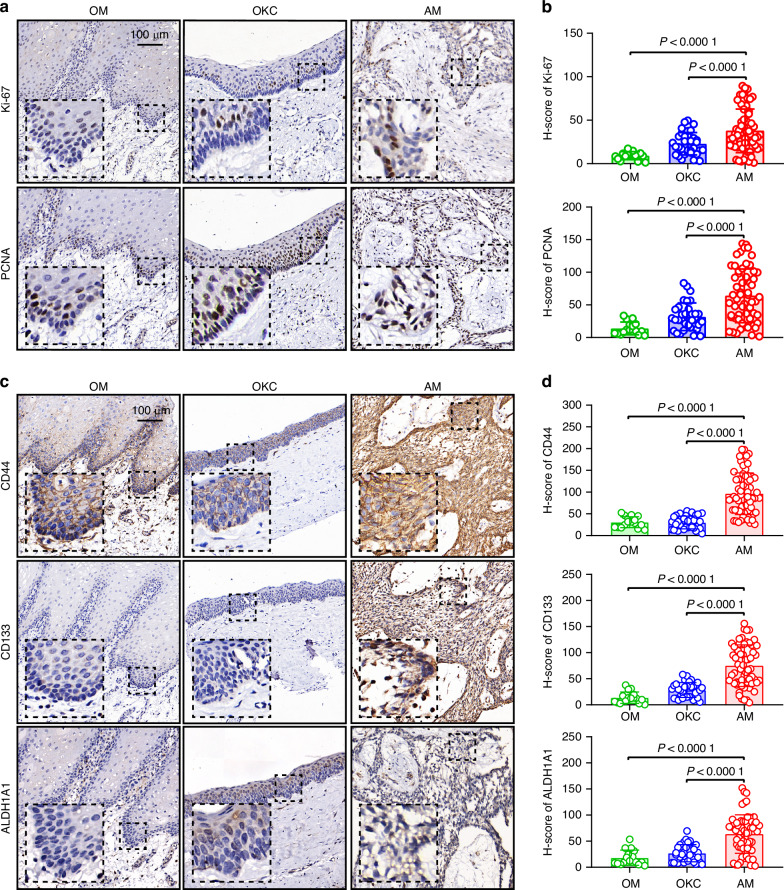
The expression of proliferation-, stemness-, and p-EMT-related proteins is increased in human ameloblastoma. **a** Representative IHC images of proliferation markers (Ki-67 and PCNA) in OM (*n* = 16), OKC (*n* = 33), and AM (*n* = 60) tissues. Scale bar, 100 µm. **b** Quantification of H-scores for the proliferation markers Ki-67 and PCNA. **c** Representative IHC images of stemness markers (CD44, CD133, and ALDH1A1) in OM (*n* = 16), OKC (*n* = 33), and AM (*n* = 60) tissues. Scale bar, 100 µm. **d** Quantification of H-scores for the stemness markers CD44, CD133, and ALDH1A1. The data are presented as the means ± SDs. Statistical significance was assessed via two-tailed unpaired Student’s *t* test (**b,** **d**)

To assess the proliferation, stemness, and p-EMT activities of AM cells, we compared the expression levels of related proteins in OM, OKC, and AM tissues (Fig. S6a). Our results revealed significantly greater expression of Ki-67 and proliferating cell nuclear antigen (PCNA), which are markers associated with cell proliferation, in AM tissues than in OM and OKC tissues (Fig. 4a, b). These findings indicate that AMs have a greater proliferative capacity. Additionally, stemness markers such as CD44, CD133, and ALDH1A1 were highly expressed in AM tissues, suggesting a pronounced stem cell-like phenotype (Fig. 4c, d). Furthermore, we assessed the expression of p-EMT markers, including LAMB3, LAMC2, and PDPN (Fig. S7a, b). These proteins were markedly upregulated in AM tissues, indicating that a partial EMT phenotype likely contributes to the invasive behavior of AMs.

To further validate these findings, we first evaluated the mRNA levels of Ki-67, PCNA, CD44, CD133, LAMB3, and LAMC2 in hTERT^+^-AM and HOK cells. The results supported the observed upregulation of proliferation, stemness, and p-EMT markers in AM tissues (Fig. S6b) and were further corroborated by western blotting (Fig. S6c). Subsequently, cell immunofluorescence (IF) staining was performed on hTERT^+^-AM and HOK cells. The results revealed significantly higher levels of Ki-67, PCNA, CD44, CD133, LAMC2 and LAMB3 in hTERT^+^-AM cells than in HOK cells (Fig. S6d, e). Collectively, these data demonstrate that AM cells exhibit high levels of proliferation, stemness, and p-EMT-related protein expression, which likely contribute to AM tumor growth and recurrence.

### PD-L1 promotes the expression of proteins related to proliferation, stemness, and partial epithelial‒mesenchymal transition

To clarify how PD-L1 regulates the expression of proteins associated with proliferation, stemness, and p-EMT in AM, we conducted a comprehensive analysis. Initially, we assessed the correlation between the expression levels of PD-L1 and those of Ki-67, PCNA, CD44, CD133, LAMB3, and LAMC2 in human AM tissues. Our results revealed positive associations between PD-L1 expression and the expression of these proliferation-, stemness-, and p-EMT-related proteins (Fig. S8a, b).

To further explore the coexpression patterns, we performed multiplex immunohistochemical (mIHC) staining of AM tissues. This analysis revealed that PD-L1 colocalized with Ki-67 and PCNA. Moreover, AM tissues with high PD-L1 expression presented higher levels of Ki-67 and PCNA than those with low PD-L1 expression did, indicating an association between PD-L1 expression and cell proliferation (Fig. S9a, b). Similar colocalization patterns were observed for PD-L1 with markers of stemness (CD44, CD133) (Fig. S9c, d) and p-EMT (PDPN, LAMC2) (Fig. S9e, f), as these markers were expressed at notably higher expression levels in tissues with high PD-L1 levels. These findings indicate that PD-L1 may play a pivotal role in enhancing the expression of proteins related to proliferation, stemness, and p-EMT in AM tissues.

**Fig. 5:**
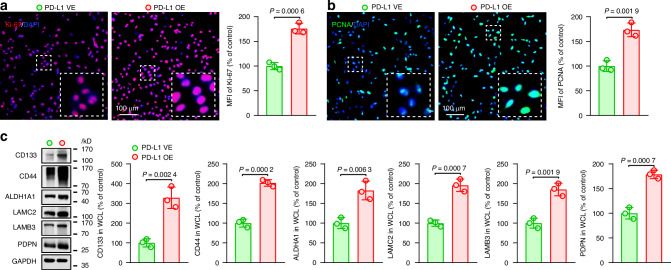
PD-L1 regulates the expression of proteins related to proliferation, stemness, and p-EMT. **a**, **b** Representative immunofluorescence staining was performed to assess the expression levels of Ki-67 (**a**) and PCNA (**b**) in hTERT^+^-AM cells with either the PD-L1 vector (PD-L1-VE) or the PD-L1 overexpression (PD-L1-OE) vector (left). The quantification of the mean fluorescence intensity (MFI) for both Ki-67 and PCNA provided insights into the proliferative status of these cells (right). **c** Western blot analysis of stemness- and p-EMT-related protein expression was performed on hTERT^+^-AM cells transfected with either the PD-L1-OE or PD-L1-VE construct. The data are presented as the means ± SDs. Statistical significance was assessed via two-tailed unpaired Student’s *t* test (**a**–**c**)

To validate these observations at the cellular level, we examined the effect of PD-L1 regulation on the expression of these proteins in hTERT^+^-AM cells. Cellular immunofluorescence analysis confirmed that PD-L1-OE hTERT^+^-AM cells presented stronger expression of Ki-67 and PCNA than control cells. Conversely, knockdown of PD-L1 resulted in downregulation of Ki-67 and PCNA expression (Fig. 5a, b and S10a, b). Similarly, compared to control cells, PD-L1-OE cells presented increased levels of CD44, CD133, ALDH1A1, LAMC2, LAMB3, and PDPN, whereas PD-L1-knockdown cells presented decreased expression of these stemness- and p-EMT-related proteins (Fig. 5c and S10c). In summary, our findings demonstrate that PD-L1 positively regulates the expression of proteins associated with proliferation, stemness, and p-EMT in hTERT^+^-AM cells, thereby enhancing their aggressive phenotype.

### PD-L1 promotes the expression of proteins related to proliferation, stemness and partial epithelial‒mesenchymal by activating the PI3K-AKT-mTOR signaling pathway

**Fig. 6:**
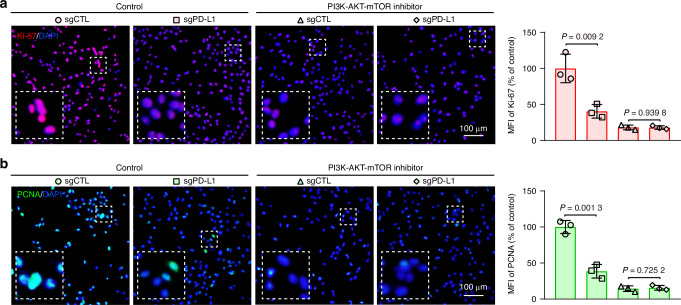
PD-L1 regulates proliferation-associated protein expression via activation of the PI3K-AKT-mTOR signaling axis. **a**, **b** Representative immunofluorescence staining was performed to assess the expression levels of Ki-67 (**a**) and PCNA (**b**) in hTERT^+^-AM cells subjected to the indicated treatments. Quantification of the mean fluorescence intensity (MFI) of Ki-67 and PCNA. The data are presented as the means ± SDs. Statistical significance was assessed via two-tailed unpaired Student’s *t* test (**a**, **b**)

Previous studies have revealed that the PD-L1-PI3K-AKT-mTOR signaling axis is involved in tumorigenesis, progression, and a poor prognosis in various tumors.^21–25^ In this study, hallmark pathway analysis revealed the involvement of AM epithelial cells with high PD-L1 expression in the PI3K-AKT-mTOR signaling pathway (Fig. 3d). This observation was validated by immunohistochemistry (IHC) of AM tissues, which revealed a positive correlation between PD-L1 expression levels and phosphorylated (p)-AKT and p-mTOR levels (Fig. S11a, b). Specifically, tissues exhibiting high PD-L1 expression presented increased levels of p-AKT and p-mTOR compared with their counterparts with low PD-L1 expression, thereby reinforcing the functional significance of this signaling axis in AM. To further validate whether the PD-L1-PI3K-AKT-mTOR signaling pathway contributes to proliferation, stemness and p-EMT potential in AM, we treated sgPD-L1 hTERT^+^-AM cells with PI3K-AKT-mTOR inhibitors. Immunofluorescence (IF) revealed a decrease in the expression levels of the proliferation markers Ki-67 and PCNA in sgPD-L1 hTERT^+^-AM cells compared with those in control cells (Fig. 6a, b). Furthermore, inhibition of AKT and mTOR phosphorylation with these inhibitors abolished the decreases in the levels of Ki-67 and PCNA in sgPD-L1 hTERT^+^-AM cells. Western blotting analysis of sgPD-L1 hTERT^+^-AM cells revealed decreased levels of p-AKT and p-mTOR; the stemness-related proteins CD44 and CD133; and the p-EMT-associated proteins LAMB3 and LAMC2. Importantly, inhibition of AKT and mTOR phosphorylation with the corresponding inhibitors abolished these decreases in protein levels (Fig. S12a, b). Collectively, these results demonstrate that PD-L1 promotes proliferation-, stemness-, and p-EMT-related protein expression in hTERT^+^-AM cells by activating the PI3K-AKT-mTOR signaling pathway.

### The PD-L1-PI3K-AKT-mTOR signaling pathway is a novel therapeutic target in ameloblastoma

**Fig. 7:**
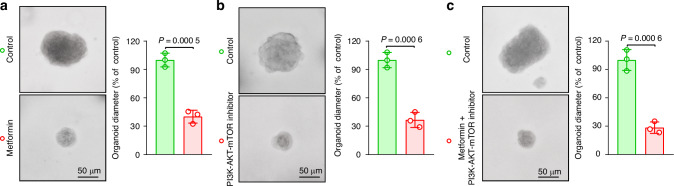
**PD-L1 is a novel therapeutic target for ameloblastoma.** **a**–**c** Representative microscopy images and quantitative analysis of the size alterations in ameloblastoma patient-derived organoids (APDOs) treated with or without a PD-L1 inhibitor (**a**), a PI3K-AKT-mTOR inhibitor (**b**), or a combination of both inhibitors (**c**). The scale bar indicates 50 μm. Statistical significance was assessed via a two-tailed unpaired Student’s *t* test for all comparisons (**a**–**c**)

Our meticulous research endeavors have revealed a pivotal role of PD-L1 in modulating the increased proliferative potential, stem-like features, and p-EMT phenotype of AM cells via the intricate PI3K‒AKT‒mTOR signaling axis. This finding underscores the potential of the PD-L1-PI3K-AKT-mTOR signaling pathway as a promising target for AM treatment. To validate this hypothesis, we conducted a comprehensive study involving the cultivation, rigorous validation, and subsequent treatment of ameloblastoma patient-derived organoids (APDOs) with either a PD-L1 inhibitor, a PI3K-AKT-mTOR inhibitor, or a combination of both. Our findings revealed that the pharmacological suppression of PD-L1 or the PI3K-AKT-mTOR pathway, alone (Fig. 7a, b) or in combination (Fig. 7c), significantly suppressed the formation and expansion of organoids derived from AM patients. Moreover, our immunofluorescence staining analyses revealed a marked decrease in the expression of the proliferative marker Ki-67 (Fig. S13a), the stemness-associated protein CD133 (Fig. S13b), and the p-EMT indicator LAMC2 (Fig. S13c) in APDOs subjected to pharmacological inhibition of the PD-L1-PI3K-AKT-mTOR pathway. These results not only affirm the intricate interplay between PD-L1 and the PI3K-AKT-mTOR signaling cascade in driving AM progression but also underscore the efficacy of targeting this axis as a therapeutic strategy. In summary, targeted inhibitors of the PD-L1-PI3K-AKT-mTOR pathway in our APDOs exhibited remarkable efficacy in impeding tumor growth, thereby positioning this pathway as a promising therapeutic target for this aggressive malignancy.

## Discussion

Ameloblastoma (AM), similar to oral squamous cell carcinoma (OSCC), is a typical epithelium-derived tumor with clinical characteristics of local invasiveness and a high recurrence rate.^26^ Research has confirmed that PD-L1 is widely expressed in OSCC tissues.^27,28^ On the one hand, membrane PD-L1 mediates immune evasion by binding to CD8 + T cells. Thus, targeting the PD-L1/PD-1 signaling axis yields benefits for OSCC patients; however, only 15%–20% of patients respond to anti-PD-1 therapy.^29,30^ Increasing research indicates that intrinsic PD-L1 mediates tumor resistance to anti-PD-1 therapy by regulating tumor cell metabolism^31^ and stemness.^32^ Notably, PD-L1 promotes metabolic competition by increasing tumor cell glucose uptake while limiting glucose availability to T cells, thereby suppressing T-cell function and facilitating tumor progression.^31^ Additionally, PD-L1 can enhance tumor stemness by driving tumor-initiating cell (TIC) formation, increasing self-renewal capacity, and conferring resistance to immune attacks, which contributes to tumor recurrence and therapy resistance.^32^ Previous studies have reported high levels of PD-L1 expression in AM tissues, but its role and mechanism in AM remain unclear. In this study, we first revealed that intrinsic PD-L1 mediates the growth and recurrence of AM by regulating proliferation, stemness, and partial epithelial‒mesenchymal transition (p-EMT) via the PI3K‒AKT‒mTOR pathway.

Stemness is the ability of cells to self-renew and differentiate into various cell types, a characteristic typically associated with stem cells. In malignant tumors, cancer stem cells can mediate tumor development and growth and are highly correlated with metastasis, treatment resistance, and recurrence, enhancing tumor heterogeneity and environmental adaptability. CD44, CD133, and ALDH1A1 are widely used as stem cell markers and play critical roles in promoting tumor growth, invasion, and recurrence. CD44 enhances invasive phenotypes by regulating tumor cell adhesion, migration, and interactions with the extracellular matrix.^33^ CD133, a marker closely associated with cancer stem cell properties, promotes tumor recurrence by maintaining self-renewal capabilities and increasing resistance to chemotherapy and radiotherapy.^34^ ALDH1A1 sustains cancer stem cell survival and proliferation by modulating reactive oxygen species (ROS) metabolism and retinoic acid signaling while also driving drug resistance and recurrence.^35^ Increasing research indicates that stemness-related proteins may play a significant role in the progression of benign jawbone tumors. Eleni-Marina Kalogirou et al. reported the upregulation of various embryonic stem cell markers (EPHA1 and SCNN1A) and detected the expression of SOX2 in the epithelium of odontogenic keratocysts (OKCs).^36^ Additionally, studies have shown that stemness markers such as CD44, SOX2, and OCT-4 are overexpressed in the epithelial components of AMs, although their regulatory mechanisms remain unexplored.^37–39^ Gan Xiong et al. classified AM cells into five subgroups at single-cell resolution and reported that the cell cycle subgroup contained cells exhibited stemness features and contributed to tumor recurrence.^40^ These findings indicate the significant impact of stemness on tumor biological activity. Similarly, in our study, higher levels of these stemness markers were observed in AM tissues than in OKC and OM tissues. When the expression of PD-L1 was upregulated or decreased in hTERT^+^-AM cells, we observed corresponding changes in the expression of stemness-related proteins. We further revealed that PD-L1, an upstream key factor, regulates stemness, thereby influencing the self-renewal, migration, and invasion abilities of hTERT^+^-AM cells.

Epithelial‒mesenchymal transition (EMT) is a biological process in which epithelial cells lose their cell polarity and adhesion ability, resulting in the acquisition of mesenchymal traits.^41^ EMT has been confirmed to be closely associated with tumor initiation, progression, and metastasis. The association between EMT and the acquisition of stem-like characteristics has been documented in several cancers, including breast, pancreatic, and colon cancers.^42^ Recent studies have revealed that cancer cells can exhibit a mixture of epithelial and mesenchymal characteristics.^43,44^ Research on AM has revealed that EMT plays a critical role in both tooth development and tumor invasion. Jie Zhang et al. revealed that interleukin-8 (IL-8) drives the EMT process by activating β-catenin and its downstream transcription factor ZEB1.^45^ Similarly, Chunmiao Jiang et al. demonstrated that IL-6 secreted by ameloblastoma-derived mesenchymal stromal cells (AM-MSCs) induces EMT in ameloblastoma epithelial cells (AM-EpiCs), promoting the formation of tumor stem-like cells and contributing to the pathogenesis and progression of this locally invasive tumor.^46^ Partial EMT (p-EMT) represents an intermediate hybrid state with both epithelial and mesenchymal phenotypes,^47^ enabling tumor cells to gain stronger migratory abilities. However, Chong Huat Siar reported that although mesenchymal markers such as a-SMA, osteonectin, and N-cadherin are expressed, AM tumor cells largely retain their epithelial morphology, indicating the presence of intermediate hybrid phenotypes with both epithelial and mesenchymal characteristics.^48^ Our study is the first to definitively establish the presence of a p-EMT state in AM. Furthermore, we revealed that PD-L1 is a pivotal regulatory protein that governs the p-EMT process in AM.

To date, traditional treatments for AM lead to high recurrence rates,^3–5^ and repeated curettage in the same area can lead to malignant transformation. Lesion excision reduces the recurrence rate to approximately 10%, but it causes facial deformities and significant physical and psychological trauma to patients due to bone grafting. Recent advances in understanding the molecular pathogenesis of AM have highlighted the use of BRAF inhibitors as a novel therapeutic approach. In a study on neoadjuvant BRAF-targeted therapy, 11 patients received dabrafenib or dabrafenib with trametinib, and all these patients achieved radiological responses and subsequently underwent successful mandible preservation surgery.^49^ While neoadjuvant BRAF inhibition holds promising potential as an organ-sparing treatment strategy for AM, its clinical application is hindered by limitations such as the acquisition of resistance and adverse effects.^50–52^ These observations underscore the need to explore alternative molecular targets that may regulate the biological behavior of AM. Our groundbreaking research reveals the critical role of intrinsic PD-L1 in modulating the growth and recurrence patterns of AM. Specifically, we demonstrated that PD-L1 exerts its effects by regulating cell stemness, proliferation, and p-EMT via the PI3K-AKT-mTOR signaling pathway. Notably, targeted inhibition of the PD-L1-PI3K-AKT-mTOR signaling axis in AM patient-derived organoids significantly attenuated tumor growth, indicating the therapeutic potential of PD-L1 blockade as a novel and promising approach for the treatment of AM. These findings not only improve our understanding of the molecular mechanisms underlying AM progression but also reveal new opportunities for the development of targeted therapies that may disrupt these oncogenic signaling cascades.

## Methods

### Tissue sample collection

The study received ethical approval from the Medical Ethical Committees of the Hospital of Stomatology at Wuhan University. Informed consent was obtained from all participants. Specimens of human oral mucosa, odontogenic keratocysts, and ameloblastomas were collected from patients at the Department of Oral & Maxillofacial Head and Neck Oncology, School and Hospital of Stomatology, Wuhan University. Each diagnosis was validated by two independent pathologists, including a board-certified oral and maxillofacial pathologist, in accordance with the 2017 WHO classification guidelines for odontogenic tumors. Recurrence was defined as development of a tumor at the same site after surgery, with pathological results confirming it as AM. The detailed clinicopathological characteristics of the AM patients are presented in Table S1, and those of the OKC patients are shown in Table S2.

### RNA extraction and RT-qPCR analysis

Total RNA was carefully extracted from three oral mucosa clinical samples and three AM clinical samples using the RNeasy Mini Kit (Qiagen, Carlsbad, CA, USA). To ensure the integrity and purity of the RNA, rigorous quality control measures were implemented. The extracted total RNA samples (2 μg per sample) were then converted into complementary DNA (cDNA) using the PrimeScript First-strand cDNA Synthesis Kit (Takara, Otsu, Japan) following the manufacturer’s protocol. One-fifth of the synthesized cDNA was subsequently utilized for quantitative polymerase chain reaction (qPCR) analysis. qPCR was performed using FastStart Universal SYBR Green Master Mix (Roche, Basel, Switzerland) on a 7900HT Fast Real-Time PCR System (Applied Biosystems, Carlsbad, CA, USA). This system offers high sensitivity and reproducibility for accurate quantification of gene expression. The primer sequences utilized for the RT‒qPCRs were specifically designed to target the genes of interest. These primer sequences were selected on the basis of their specificity, efficiency, and ability to generate reliable results. The sequences of primers used were as follows: *MKI67*: 5′-ACGCCTGGTTACTATCAAAAGG-3′ and 5′-CAGACCCATTTACTTGTGTTGGA-3′, *PCNA*: 5′-CCTGCTGGGATATTAGCTCCA-3′ and 5′-CAGCGGTAGGTGTCGAAGC-3′, *CD44*: 5′-CTGCCGCTTTGCAGGTGTA-3′ and 5′-CATTGTGGGCAAGGTGCTATT-3′, *CD133*: 5′-AGTCGGAAACTGGCAGATAGC-3′ and 5′-GGTAGTGTTGTACTGGGCCAAT-3′, *LAMB3*: 5′-GCAGCCTCACAACTACTACAG-3′ and 5′-CCAGGTCTTACCGAAGTCTGA-3′, *LAMC2*: 5′- GACAAACTGGTAATGGATTCCGC-3′ and 5′- TTCTCTGTGCCGGTAAAAGCC-3′. GAPDH served as the internal control for normalization to accurately quantify gene expression. Target mRNA levels were assessed using CT values with GAPDH as the reference gene. The 2^−∆∆CT^ method was applied to calculate relative mRNA quantities, providing reliable fold-change estimates.

### Cell culture and treatments

Human oral keratinocytes (HOKs) were obtained from the American Type Culture Collection (ATCC). The immortalized hTERT^+^-AM cell line was graciously provided by Professor Qian Tao of Sun Yat-sen Memorial Hospital. These hTERT^+^ -AM cells were maintained in DMEM (Thermo Fisher Scientific, #C11995500BT) supplemented with 10% fetal bovine serum (FBS) (Thermo Fisher Scientific, #A3160801) and 1% penicillin/streptomycin (Thermo Fisher Scientific, #15140-122). All the cell cultures were incubated at 37 °C in a humidified atmosphere with 5% CO_2_ until experiments were performed.

To suppress PD-L1 expression in hTERT^+^-AM cells, 50 µmol/L metformin was used. To inhibit the PI3K-AKT-mTOR signaling pathway, a combination of 50 mmol/L LY294002 and 100 nmol/L rapamycin (both sourced from EMD Calbiochem-Millipore, USA) was utilized.

### Single-cell RNA sequencing

Following surgical resection, two fresh tissue samples were preserved in tissue preservation solution (2–8 °C) (Singleron Biotechnologies, China) and quickly transported to the laboratory. Single-cell suspensions were prepared and loaded onto microfluidic chips. Using the GEXSCOPE® Single-Cell RNA Library Kit (Singleron Biotechnologies), scRNA-seq libraries were constructed. Each library, prepared at 4 nmol/L, was pooled and sequenced on the Illumina HiSeq X platform with 150 bp paired-end reads.

### ScRNA-seq data quality control, processing, and cell type identification

We utilized Seurat (v4.0.0) in R (v4.0.2) to process each sample’s gene-barcode expression matrix. Quality control measures included filtering out cells with fewer than 200 or more than 9 000 expressed genes, cells with over 10% mitochondrial gene content, and those with more than 7% hemoglobin gene content. Ribosomal and mitochondrial genes were subsequently removed from the dataset. To ensure data integrity, potential doublets were identified and excluded using DoubletFinder (v2.0.3).

Normalization and variance stabilization of the data were performed using sctransform (v0.3.2). Following these preprocessing steps, the samples were integrated into a single Seurat object using the IntegrateData function. For dimensionality reduction, we applied principal component analysis (PCA) to the top 3 000 highly variable genes (HVGs). The ElbowPlot function was employed to identify the principal components that significantly contributed to the variance in the data. Cell type assignment was performed via Seurat’s FindAllMarkers function to identify differentially expressed genes (DEGs) with default parameters. Cluster annotation was conducted on the basis of established marker genes from the literature. Specifically, we identified the following cell types: tumor epithelial cells expressing the markers *KRT14*, *KRT15*, *KRT19*, and *KRT6A*; myeloid cells characterized by *CD74*, *HLA-DRA*, *HLA-DB1*, and *LYZ* expression; endothelial cells characterized by *PECAM1*, *ENG*, and *VWF* expression; and fibroblasts characterized by *COL3A1*, *COL1A1*, *COL1A2*, and *LUM* expression.

### Western blotting

Western blotting was conducted following established protocols.^53,54^ In brief, proteins were extracted from both tissues and cultured cells. The total protein concentration was measured via a BCA Assay Kit. Proteins were then separated by SDS‒polyacrylamide gel electrophoresis and transferred onto polyvinylidene fluoride (PVDF) membranes. The membranes were blocked with 5% nonfat milk for 1 h at room temperature. Following blocking, the membranes were incubated overnight at 4 °C with primary antibodies. The next day, the membranes were incubated with HRP-conjugated secondary antibodies for 1 h at room temperature. Detection was carried out using enhanced chemiluminescence (ECL) Western blotting detection reagents. The following primary antibodies were utilized in the study: anti-human PD-L1 (Cell Signaling Technology, #13684), anti-human CD44 (Cell Signaling Technology, #3570), anti-human/mouse CD133 (Proteintech, #18470-1-AP), anti-human/mouse ALDH1A1 (Proteintech, #60171-1-Ig), anti-human LAMC2 (Abcam, #ab210959), anti-human LAMB3 (Abcam, #ab97765), anti-human PDPN (Cell Signaling Technology, #9047S), and anti-human/mouse GAPDH (ABclonal, #AC002).

### Immunohistochemistry and H&E staining

Detailed immunohistochemistry (IHC) procedures were performed as previously described.^55^ In summary, the OM, OKC, and AM tissues were fixed in 4% paraformaldehyde overnight, embedded in paraffin, and sectioned into 4 µm thick slices. Immunohistochemical staining was carried out according to the protocols specified in the immunohistochemical kit. Chromogenic development was achieved via the use of diaminobenzidine (DAB), and counterstaining was performed with hematoxylin.

High-resolution images of the stained slides were captured via a Panoramic Scanner (3DHISTECH) equipped with background subtraction capabilities. Both cellular and membranous staining, along with cell counts in selected regions across all tissue microarray (TMA) tissues, were analyzed via CaseViewer software (3DHISTECH) and NDP.view2 software (Hamamatsu). To quantify the staining, a histoscore (H score) for each tissue core was calculated. This score was determined by assessing the percentage of cells exhibiting positive staining according to the following formula: (percentage of strong positive staining) × 3 + (percentage of moderate positive staining) × 2 + (percentage of weak po'sitive staining) × 1). The following primary antibodies were used: anti-PD-L1 (Cell Signaling Technology, #29122), anti-Ki-67 (ZSGB-BIO, #ZM-0166), anti-PCNA (Cell Signaling Technology, #2586), anti-CD44 (Cell Signaling Technology, #3570), anti-CD133 (Proteintech, #18470-1-AP), anti-ALDH1A1 (Proteintech, #60171-1-Ig), anti-LAMC2 (Abcam, #ab210959), anti-LAMB3 (Abcam, #ab97765), and anti-PDPN (Cell Signaling Technology, #9047S).

For the H&E assay, paraffin-embedded tumor sections were stained with an H&E kit (Beyotime, # C0105S) following the manufacturer’s instructions.

### Cell immunofluorescence

Immunofluorescence staining was conducted on fixed cells to visualize the expression of specific proteins. The cells were initially permeabilized with 0.3% Triton X-100 and subsequently blocked in bovine serum albumin (BSA) buffer for 1 h. After blocking, the cells were incubated overnight at 4 °C with primary antibodies. This was followed by a 1 h incubation with fluorophore-conjugated secondary antibodies. Nuclear counterstaining was performed using DAPI. The primary antibodies used in this study were as follows: anti-PD-L1 (Cell Signaling Technology, #29122), anti-Ki-67 (ZSGB-BIO, #ZM-0166), anti-PCNA (Cell Signaling Technology, #2586), anti-CD44 (Cell Signaling Technology, #3570), anti-CD133 (Proteintech, #18470-1-AP), anti-LAMC2 (Abcam, #ab210959), and anti-LAMB3 (Abcam, #ab97765).

### Multiplex immunohistochemistry (mIHC)

mIHC was performed using a multiplex IHC kit according to the manufacturer’s protocols (Akoya Bioscience, #NEL801001KT).^56^ Briefly, after deparaffinization and rehydration, antigen retrieval was conducted using EDTA buffer and microwave heating. Following a 10 min block in blocking solution at room temperature, primary antibodies were incubated at 37 °C for 1 h. Secondary antibodies were incubated at room temperature for 10 min, followed by incubation with Opal fluorophores for 10 min. This process of antigen retrieval, blocking, and incubation with primary and secondary antibodies and Opal fluorophores was repeated, followed by DAPI staining for nuclear visualization. The slides were then scanned with a PerkinElmer Vectra Polaris (PerkinElmer, #Vectra3). The primary antibodies used in this study included the following: anti-PD-L1 (Cell Signaling Technology, #29122), anti-pan cytokeratin (Pan-CK) (Cell Signaling Technology, #4545), anti-Ki-67 (ZSGB-BIO, #ZM-0166), anti-PCNA (Cell Signaling Technology, #2586), anti-CD44 (Cell Signaling Technology, #3570), anti-CD133 (Proteintech, #18470-1-AP), anti-LAMC2 (Abcam, #ab210959), and anti-PDPN (Cell Signaling Technology, #9047S).

### Colony formation assay

To assess clonogenicity, the cells were plated in 6-well plates at a density of 2 000 cells per well and cultured in DMEM supplemented with 10% fetal bovine serum (FBS) for 10 to 14 days. After incubation, the colonies were fixed with 4% paraformaldehyde for 15 min and subsequently stained with 0.4% crystal violet for 15 min. After thorough washing with phosphate-buffered saline (PBS) and air drying, colonies comprising more than 50 cells were counted and photographed using a microscope.

### Generation of PD-L1 knockout and overexpression cell lines

#### PD-L1 knockout in hTERT^+^ -AM cells

Stable knockout of PD-L1 in hTERT^+^-AM cells was achieved via the CRISPR/Cas9 genome editing system in accordance with established protocols. For targeted disruption of the PD-L1 gene, a guide RNA specific to human PD-L1 (target sequence: TACCGCTGCATGATCAGCTA) was cloned and inserted into the lentiviral vector pLentiCRISPRv2. This construct was obtained from GenScript. The hTERT^+^-AM cells were transduced with the recombinant lentivirus carrying the PD-L1 guide RNA. After transduction, PD-L1 knockout cells were selected using puromycin to ensure the establishment of a cell line with stable gene knockout.

#### PD-L1 overexpression in hTERT^+^-AM cells

To generate hTERT^+^ -AM cells stably overexpressing PD-L1, they were infected with lentiviruses engineered to overexpress PD-L1. The recombinant lentivirus was procured from GeneChem (Shanghai, China). Following the manufacturer’s protocol, the cells were incubated with the lentivirus for 8 h. After this incubation period, the medium was replaced with DMEM supplemented with 10% fetal bovine serum (FBS; Thermo Fisher Scientific, #A3160801) and 1% penicillin/streptomycin.

Fluorescence was observed 48 h post infection, indicating successful transduction. To establish stable overexpression, the cells were then cultured in the presence of 4 µg/mL puromycin for 7 days.

### Validation of PD-L1 expression

The efficacy of PD-L1 knockout and overexpression in transfected hTERT^+^-AM cells was confirmed via Western blot analysis.

### Wound-healing assay

To assess the migratory ability of hTERT^+^-AM cells, a wound-healing assay was performed. hTERT^+^-AM cells were seeded in 6-well plates and cultured until they reached approximately 70%–80% confluence. A straight scratch, or “wound,” was created in the cell monolayer using a sterile pipette tip. Next, the cells were rinsed to remove debris and then cultured in serum-free DMEM. Images of the wound area were captured at designated time points to monitor and quantify the rate of cell migration into the wound space. The images were analyzed to determine the extent of wound closure over time, providing insights into the migratory behavior of the cells.

### Spheroid formation assay

To evaluate the sphere formation capacity of hTERT^+^-AM cells, a spheroid formation assay was conducted. Briefly, hTERT^+^-AM cells were seeded into ultralow attachment 6-well plates (Corning) at a density of 5 × 10^4^ cells per well. These specialized plates prevent cell adhesion to the surface, facilitating spheroid formation. The cells were cultured in serum-free medium specifically formulated to support spheroid growth. This medium was supplemented with 2% B27 supplement (Life Technologies), 100 U/mL penicillin, 100 U/mL streptomycin, 20 ng/mL human epidermal growth factor (EGF; PeproTech), and 10 ng/mL human basic fibroblast growth factor (bFGF; PeproTech). After two weeks of culture, the spheroids were observed and counted using an inverted microscope. This allowed for the assessment of spheroid formation efficiency and morphology.

### Invasion assay

To assess the invasive potential of hTERT^+^-AM cells, an invasion assay was performed using 24-well plates with 8 µm pore inserts. The hTERT^+^-AM cells were starved in serum-free DMEM for one day. The cells were resuspended in serum-free DMEM at a concentration of 5 × 10^5^ cells per mL. Each well was coated with 100 µL of 1.6 mg/mL Matrigel. A 100 µL cell suspension was added to the upper chamber, and 500 µL DMEM with 10% FBS was added to the lower chamber. After 16 h, the cells were fixed with paraformaldehyde for 15 min and stained with 0.4% crystal violet for 20 min at room temperature. After washing with PBS, the cells on the upper surface of the insert were removed with a cotton swab, and the remaining cells were air-dried, photographed, and counted under a microscope.

### EdU assay

An EdU (5-ethynyl-2’-deoxyuridine) assay was performed to assess the proliferation of hTERT^+^-AM cells. hTERT^+^-AM cells were seeded in 96-well plates at a density of 5 000 cells per well in 100 µL of medium. Following overnight culture to allow cell attachment, the cells were treated with 2× EdU working solution (20 µmol/L), which was prewarmed to 37 °C. The cells were incubated for 2 h, fixed with 4% paraformaldehyde for 15 min, washed, and permeabilized with 0.3% Triton X-100 for 15 min. After washing, the cells were incubated with Click reaction solution and then stained with Hoechst 33342 for 10 min at room temperature in the dark. Fluorescence images of the stained cells were captured via a fluorescence microscope.

### Organoid culture

To establish and maintain organoids from ameloblastoma (AM) tissues, the following protocol was followed.^40,57^ The AM tissues were minced and incubated at 37 °C with collagenase type IV (Stemcell, #07909) for 50 min. After digestion, 10 mL of DMEM/F12 (Thermo Fisher Scientific, #C11330500BT) was added to dilute the collagenase. The resulting suspension was filtered through a 100 µm sieve (Falcon, #352360) and centrifuged at 1 000 r/min for 5 min. The cell pellet was then resuspended in BioCoat MATRIGEL MATRIX (Corning, #354234) mixed with organoid medium at a 1:1 ratio. This mixture was plated onto 24-well culture plates and incubated at 37 °C for 30 min. Organoids were maintained in custom-formulated media composed of DMEM/F12 (Thermo Fisher Scientific, #C11330500BT), 1× B27 supplement (Thermo Fisher Scientific, #12587010), 1.25 mmol/L N-acetyl-L-cysteine (Sigma, #A7250), 10 mmol/L nicotinamide (Sigma, #N0636), 50 ng/mL human EGF (PeproTech, #AF-100-15), 500 nmol/L A83-01 (PeproTech, #9094360), 10 ng/mL human FGF10 (PeproTech, #100-26-5), 5 ng/mL human FGF2 (Sino Biological Inc., #10014-HNAE), 1 μmol/L prostaglandin E2 (MCE, #HY-101952), 0.3 μmol/L CHIR 99021 (Sigma, #SML1046), 1 μmol/L forskolin (Abcam, #ab120058), 50 ng/mL R-spondin (R&D Systems, #3266-RS), and 25 ng/mL Noggin (PeproTech). To facilitate organoid outgrowth, 10 μmol/L of the ROCK inhibitor Y-27632 (TargetMol, #T1725) was added to the medium after the first week. The medium was changed every 48–72 h, and the organoids were passaged every 1–2 weeks.

### Statistical analysis

Statistical analyses were performed via GraphPad Prism, version 9.3.1 (GraphPad Software). Differences between two groups were analyzed via Student’s *t* test. Survival curves were analyzed via the log-rank test. Pearson’s correlation coefficient was used for correlation analysis. A *P* value of less than 0.05 was considered to indicate statistical significance.

## Supplementary information

Revised supplementary materials
