# Supplementary material for: Programmed death-ligand 1 regulates ameloblastoma growth and recurrence
Source: Int J Oral Sci. 2025 Apr 16;17:29. doi: 10.1038/s41368-025-00364-w (PMC12003687; doi:10.1038/s41368-025-00364-w)
Supplement: Supplementary file 1 — Revised supplementary materials [file 41368_2025_364_MOESM1_ESM.pdf]

Supplementary materials

PD-L1 regulates ameloblastoma growth and recurrence

Lin-Zhou Zhang[1*], Hao Lin[1*], Jia-Jie Liang[1], Xuan-Hao Liu[1], Chen-Xi Zhang[1], Qi-Wen Man[1,2], Rui-Fang Li[1,2], Yi Zhao[1,3, #], Bing Liu[1,2, #]

[1]State Key Laboratory of Oral & Maxillofacial Reconstruction and Regeneration, Key Laboratory of Oral Biomedicine Ministry of Education, Hubei Key Laboratory of Stomatology, School & Hospital of Stomatology, Wuhan University, Wuhan 430079, China.

[2]Department of Oral and Maxillofacial Surgery, School and Hospital of Stomatology, Wuhan University, Wuhan 430079, China.

[3]Department of Prosthodontics, School and Hospital of Stomatology, Wuhan University, Wuhan, China.

[*]Co-first author

[#]Correspondence: zhao_yi@whu.edu.cn; liubing9909@whu.edu.cn

Supplementary Figure and Figure Legends

Figure S1

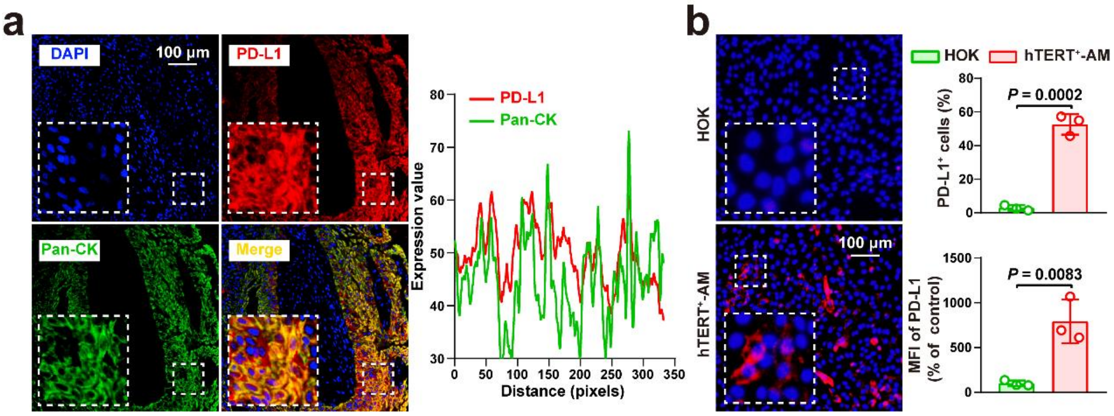

Figure S1. Immunofluorescence staining analysis of PD-L1 expression in AM cells

a Immunofluorescence staining for PD-L1 (red) and Pan-CK (green) in AM tissues. The fluorescence intensity profiles are plotted on the right. Scale bar, 100 μm. **b** Representative immunofluorescence images of PD-L1 in human oral keratinocytes (HOKs) and $hTERT^{+}$-AM cells (left). Quantification of the percentage of PD-L1 -positive HOKs and $hTERT^{+}$-AM cells and the mean fluorescence intensity (MFI) of PD-L1 in these cells (right). Scale bar, 100 μm.

Figure S2

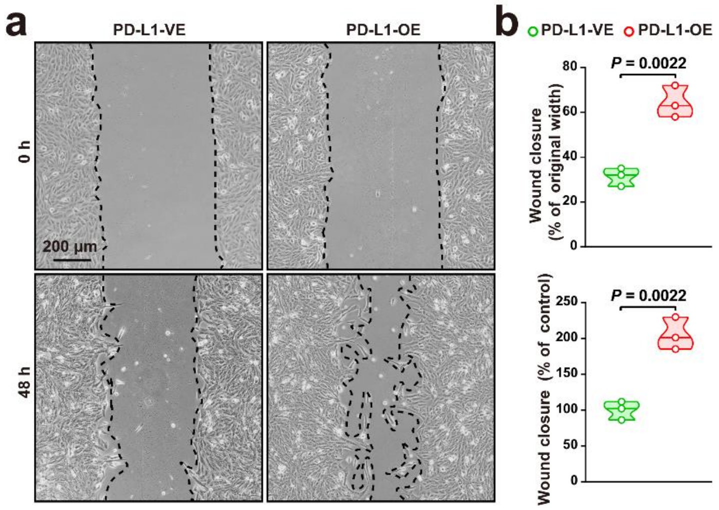

Figure S2. Evaluation of migration ability in PD-L1-VE and PD-L1-OE hTERT⁺-AM cells

a Representative images of wound healing in PD-L1-VE and PD-L1-OE hTERT⁺-AM cells at 0 h and 48 h post-scratch (left). **b** Quantitative analysis of the percentage of wound closure (right). Scale bar, 200 μm.

Figure S3

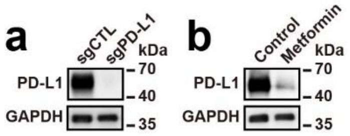

Figure S3. Western blot analysis of PD-L1 expression in hTERT⁺-AM cells following PD-L1 manipulation.

Figure S4

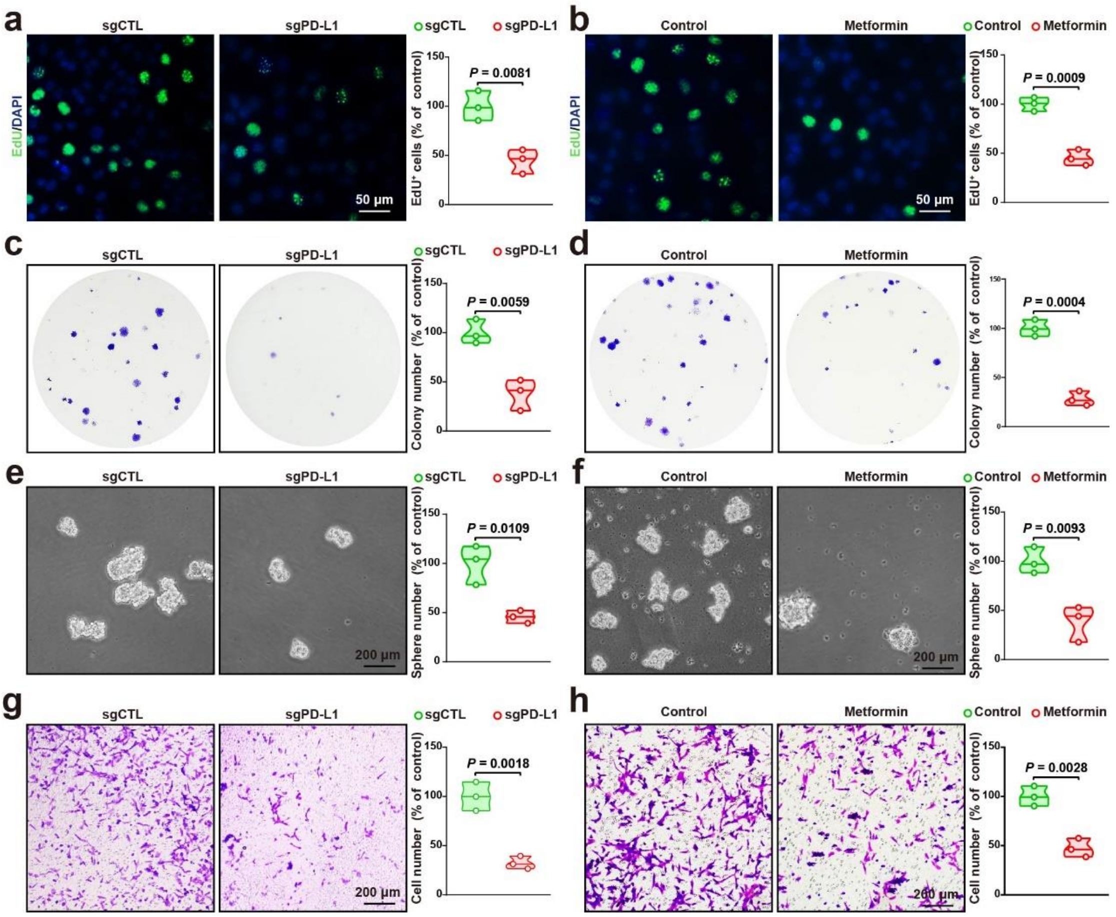

Figure S4. Targeting PD-L1 inhibits self-renewal capacity, tumorigenesis, and invasiveness in hTERT$^+$-AM cells

a Representative image of EdU staining (green) in sgControl (sgCTL) and sgPD-L1 hTERT$^+$-AM cells, with nuclei counterstained by DAPI (blue). Quantitative analysis of EdU-positive hTERT$^+$-AM cells (right). Scale bar, 50 μm. **b** Representative images of EdU staining (green) in control and metformin-treated hTERT$^+$-AM cells. Scale bar, 50 μm. **c** Representative images showing the colony formation of sgCTL and sgPD-L1 hTERT$^+$-AM cells stained with crystal violet (left). Quantitative analysis of the number of crystal violet-stained colony (right). **d** Representative images of colony formation in control and metformin-treated hTERT$^+$-AM cells stained with crystal violet. Quantitative

analysis of the number of crystal violet-stained colony (right). **e** Representative images of spheroid formation in sgCTL and sgPD-L1 $hTERT^{+}$-AM cells (left). Quantitative analysis of the number of spheres (right). Scale bar, 200 μm. **f** Representative images of spheroid formation in control and metformin-treated $hTERT^{+}$-AM cells. Quantitative analysis of the number of spheres (right). Scale bar, 200 μm. **g** Representative images showing the invasive ability of sgCTL and sgPD-L1 $hTERT^{+}$-AM cells, stained with crystal violet (left). Quantitative analysis of the number of invading $hTERT^{+}$-AM cells (right). Scale bar, 200 μm. **h** Representative images of the invasive ability of control and metformin-treated $hTERT^{+}$-AM cells, stained with crystal violet (left). Quantitative analysis of the number of invading $hTERT^{+}$-AM cells (right). Scale bar, 200 μm. Data are expressed as mean ± SD and analyzed using a two-tailed unpaired Student's t-test (**a**-**h**). All results are representative of three independent experiments.

Figure S5

a b

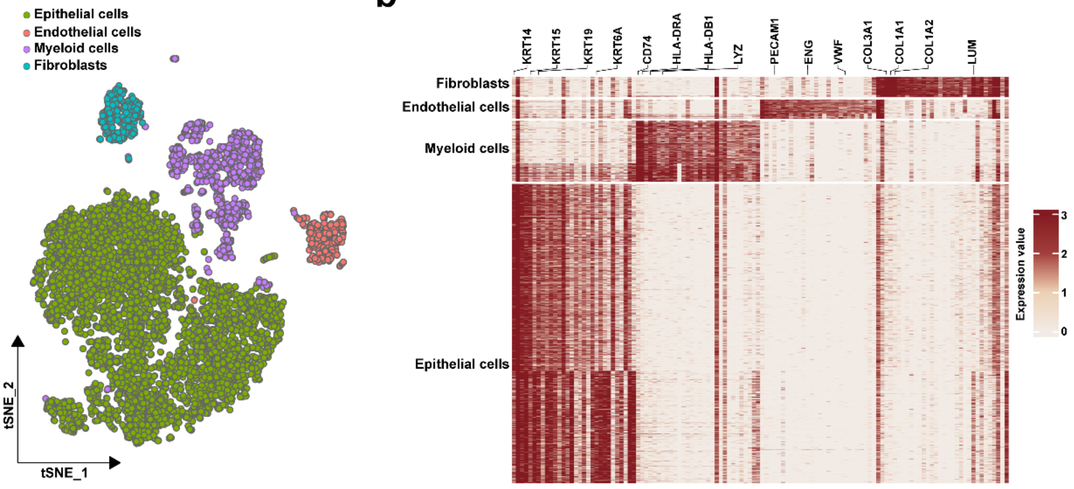

Figure S5. Single-cell RNA sequencing uncovers cell subpopulations of AM tissues

a t-SNE plot of single cells from the scRNA-seq analysis labeled by cell type. **b** Heatmap of marker genes for different cell types.

Figure S6

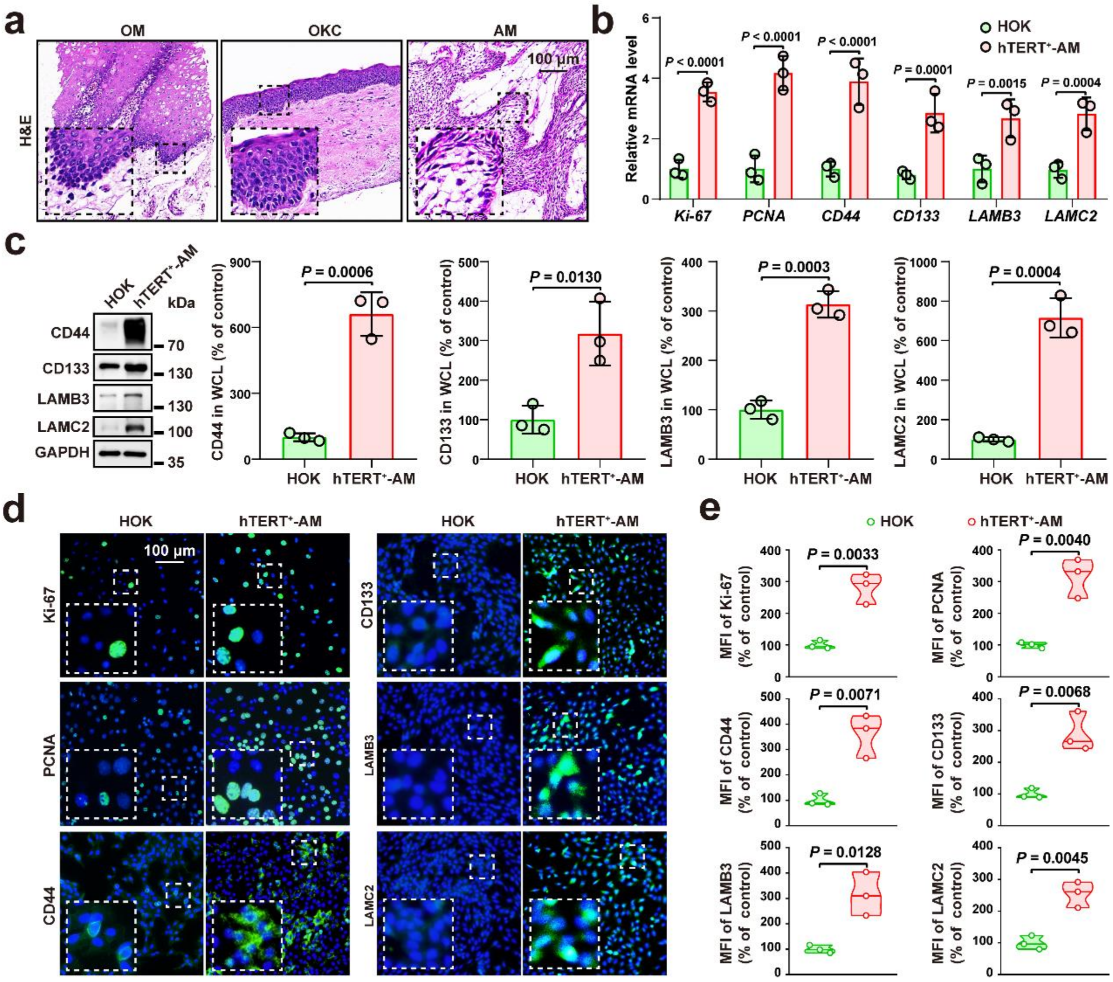

Figure S6. Increased proliferation, stemness, and p-EMT-related protein expression in ameloblastoma cells

a Representative hematoxylin and eosin (H&E) in oral mucosa (OM), odontogenic keratocyst (OKC), and AM tissues. **b** Quantification for the mRNA levels of *MKI67*, *PCNA*, *CD44*, *CD133*, *LAMB3*, and *LAMC2* in HOK and AM cells. **c** Western blot analysis of CD44, CD133, LAMB3, and LAMC2 expression in HOK and hTERT^{+}-AM cells. **d** Representative immunofluorescence staining for Ki-67, PCNA, CD44, CD133, LAMB3, and LAMC2 in HOK and hTERT^{+}-AM cells. **e** Quantification of mean fluorescence intensity (MFI) of proliferation-, stemness-, and p-EMT-related markers between HOK and hTERT^{+}-AM cells.

Data are presented as mean ± SD. Statistical significance was determined by two-way ANOVA (**b**) and two-tailed unpaired Student's t-test (**c, e**).

Figure S7

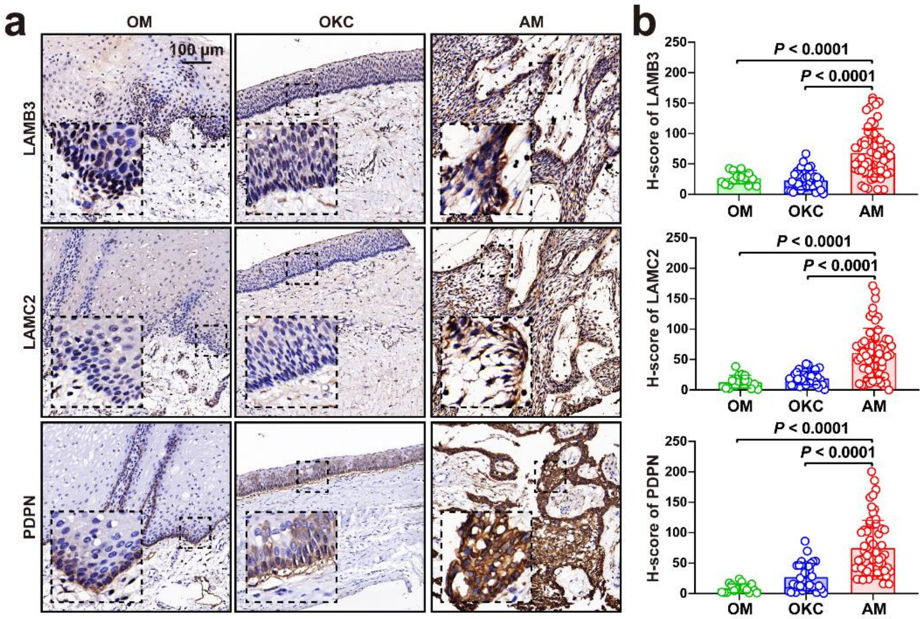

Figure S7. Enhanced expression of p-EMT-related proteins in human ameloblastoma

a Representative IHC images of p-EMT markers (LAMB3, LAMC2, and PDPN) in OM (n = 16), OKC (n = 33), and AM (n = 60) tissues. Scale bar, 100 μm. **b** Quantification of H-scores for p-EMT marker LAMB3, LAMC2, and PDPN. Data are presented as mean ± SD. Statistical significance was determined by two-tailed unpaired Student’s t-test.

Figure S8

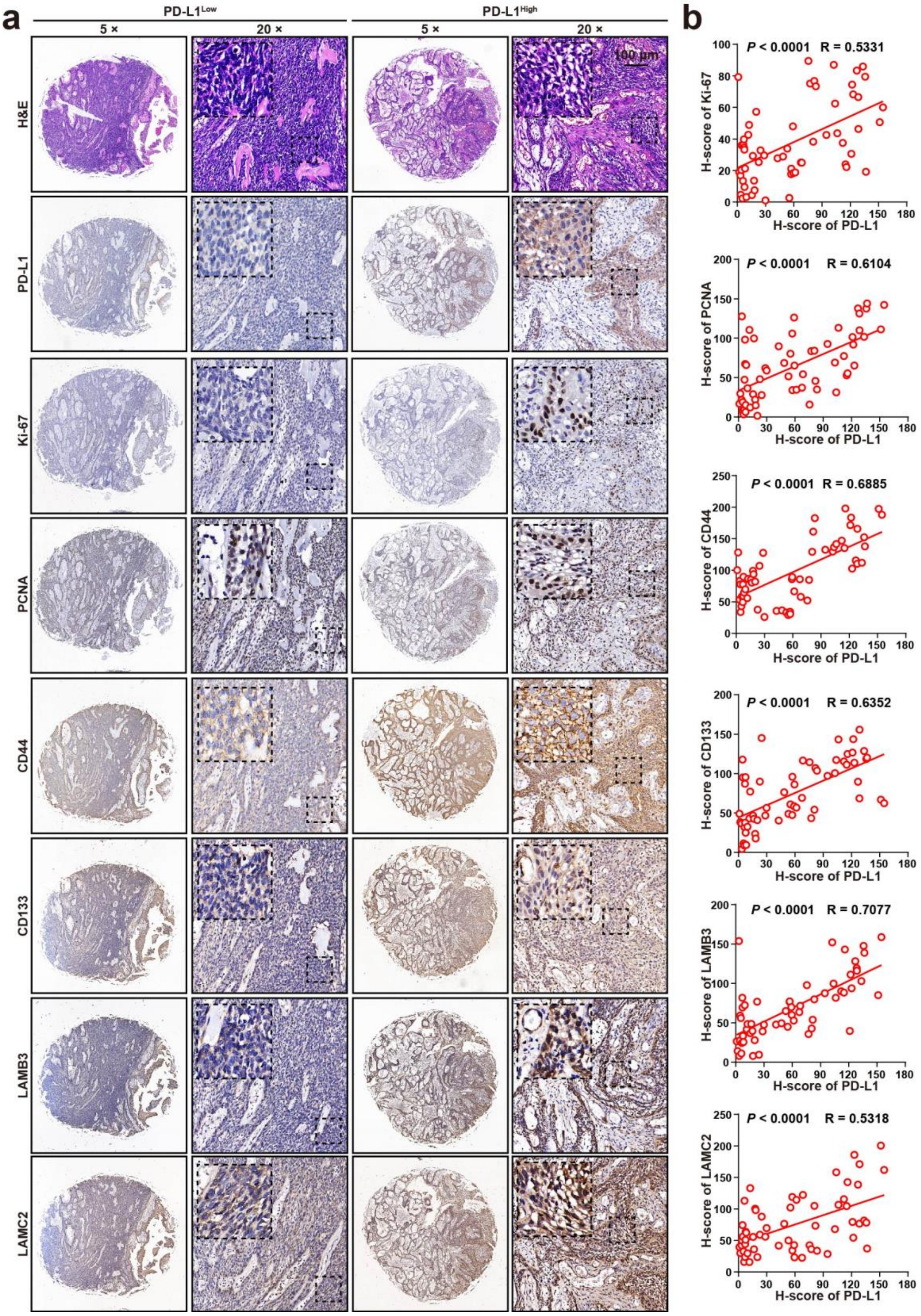

Figure S8. PD-L1 expression is positively associated with the levels of proliferation, stemness, and p-EMT-related factors in human AM patients

a Representative image of H&E, PD-L1, Ki-67, PCNA, CD44, CD133, LAMB3, and LAMC2 staining in AM tissues with low PD-L1 expression ($PD\text{-}L1^{Low}$) (n = 26) and high PD-L1 expression ($PD\text{-}L1^{High}$) (n = 34). Images are shown at 5× and 20× magnifications. Scale bar, 100 μm. **b** Pearson correlation analysis between PD-L1 and Ki-67, PCNA, CD44, CD133, LAMB3, and LAMC2 expression in AM tissues.

Figure S9

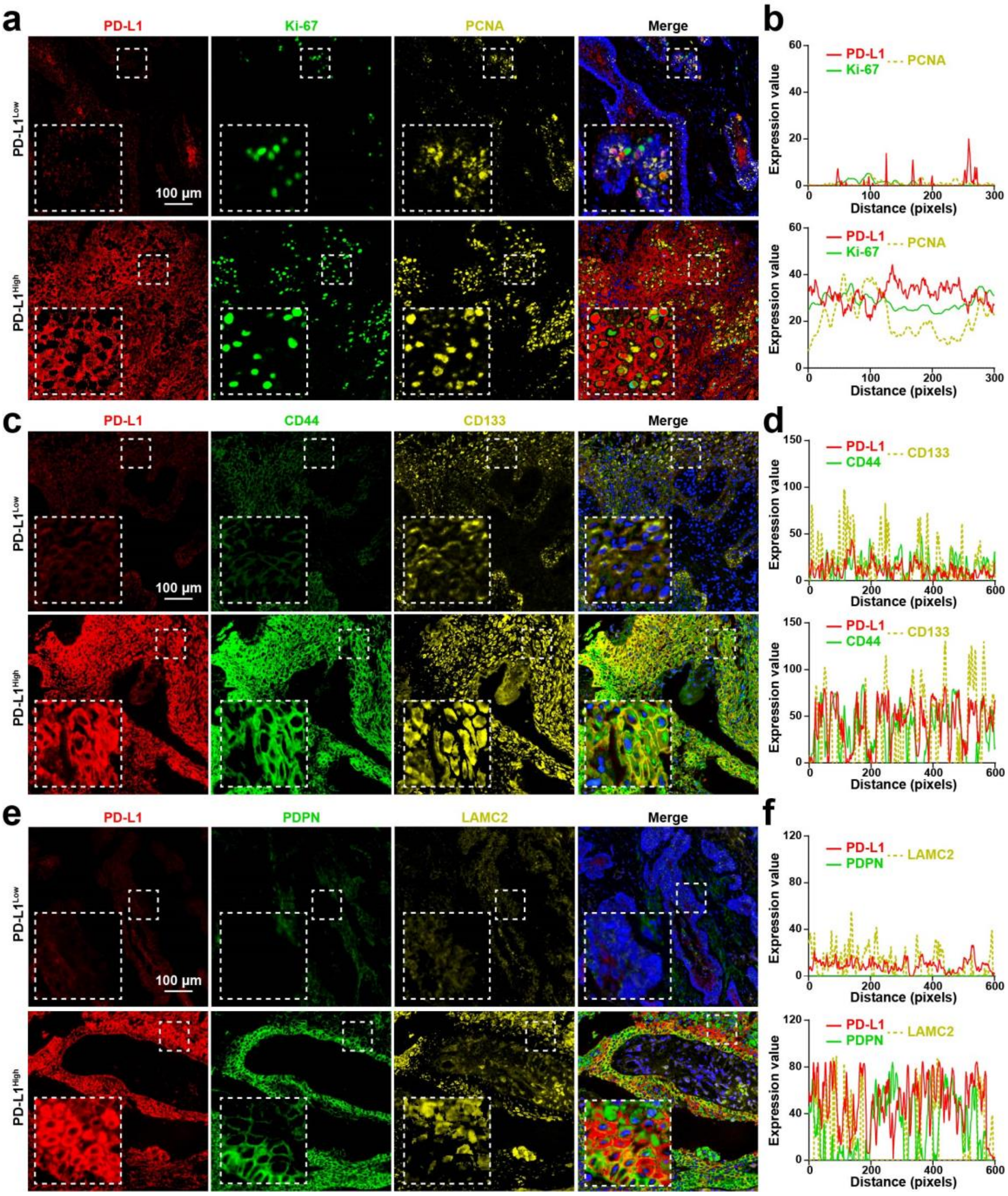

Figure S9. Co-localization of PD-L1 with proliferation, stemness, and p-EMT-related markers in human ameloblastoma

a Representative image of PD-L1 co-staining with Ki-67 or PCNA in ameloblastoma tissues with PD-L1^{Low} and PD-L1^{High} expression. Scale bar, 100 μm. **b** Quantitative analysis of the fluorescence intensity profiles reveals the co-

expression patterns of PD-L1 with Ki-67 and PCNA in ameloblastoma tissues. **c** Representative images of PD-L1 co-staining with CD44 or CD133 in ameloblastoma tissues with PD-$L1^{Low}$ and PD-$L1^{High}$ expression. Scale bar, 100 μm. **d** Quantitative analysis of the fluorescence intensity profiles highlights the co-expression patterns of PD-L1 with CD44 and CD133 in ameloblastoma tissues. **e** Representative images of PD-L1 co-staining with PDPN or LAMC2 in ameloblastoma tissues with PD-$L1^{Low}$ and PD-$L1^{High}$ expression. Scale bar, 100 μm. **f** Quantitative assessment of the fluorescence intensity profiles reveals the co-expression patterns of PD-L1 with PDPN and LAMC2 in ameloblastoma tissues.

Figure S10

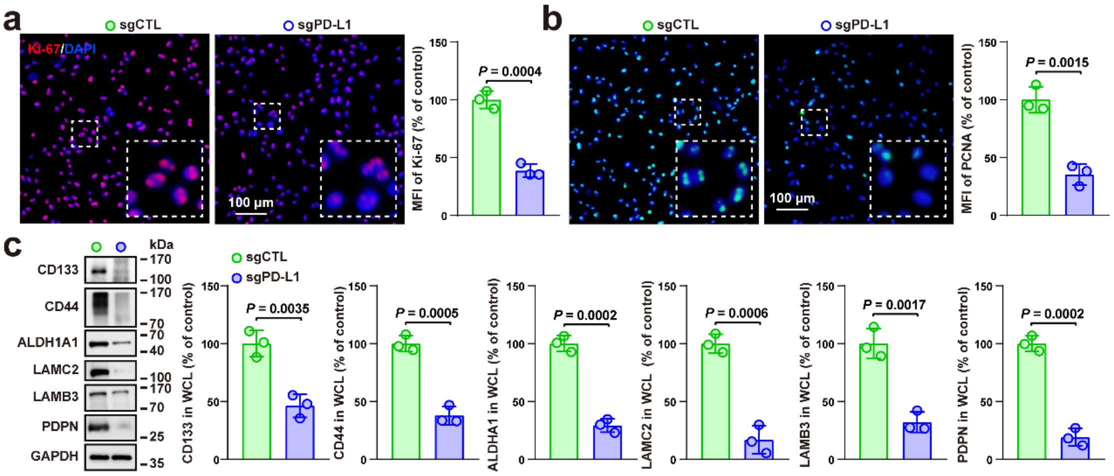

Figure S10. PD-L1 modulates the expression of molecular networks associated with proliferative capacity, cellular stemness maintenance, and p-EMT activation

a-b Immunofluorescence staining for Ki-67 (**a**) and PCNA (**b**) was conducted in hTERT$^+$-AM cells subjected to PD-L1 knockdown (sgPD-L1) and nontargeting control (sgCTL) conditions (left). The quantification of the MFI for Ki-67 and PCNA in these cells offered further comparative analysis of proliferative markers in response to PD-L1 modulation (right). **c** Western blotting for stemness- and p-EMT-related protein expression was conducted on hTERT$^+$-AM cells transfected with sgPD-L1 and sgCTL. The data are presented as the means ± SDs. Statistical significance was assessed via two-tailed unpaired Student's t test (**a-c**).

Figure S11

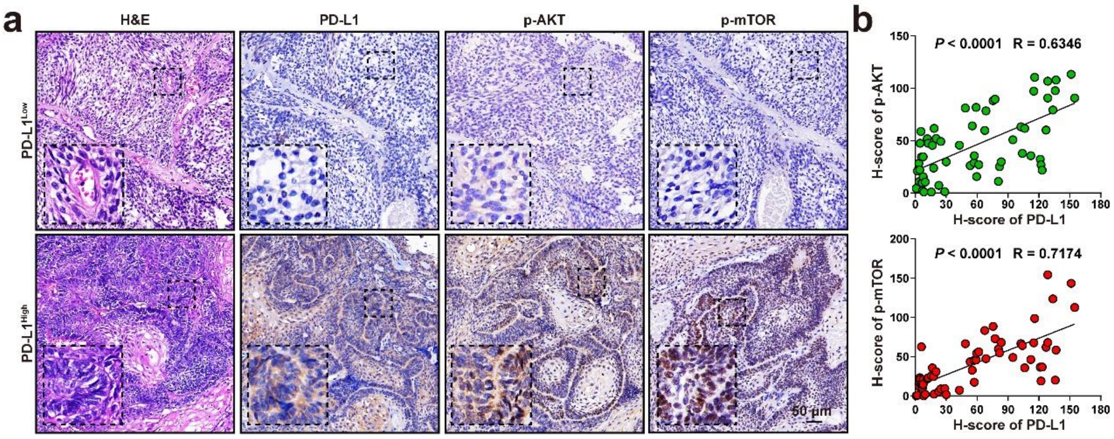

Figure S11. The expression level of PD-L1 is positively correlated with p-AKT and p-mTOR expression in human ameloblastoma

a Representative hematoxylin and eosin (H&E) and immunohistochemical (IHC) images of PD-L1, p-AKT, and p-mTOR expression in PD-L1^{Low} and PD-L1^{High} AM tissues. **b** Correlation analysis between PD-L1 levels and p-AKT and p-mTOR expression in AM tissues.

Figure S12

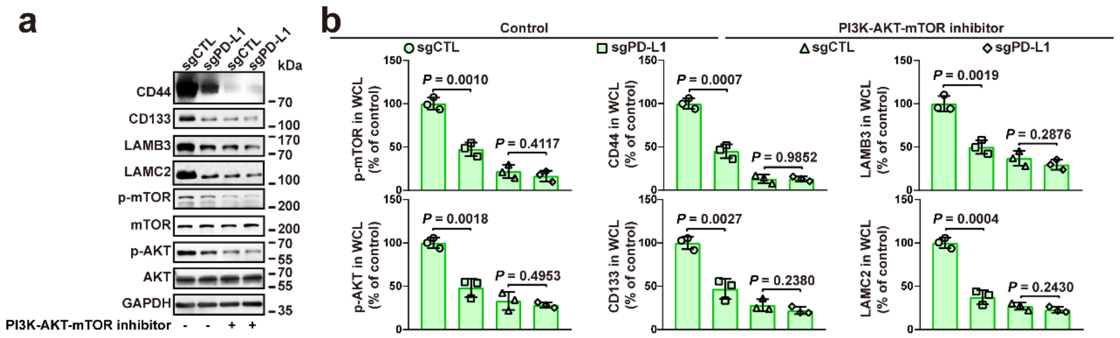

Figure S12. PD-L1 modulates the expression of proteins related to stemness and p-EMT via activation of the PI3K-AKT-mTOR signaling axis

a Western blotting for stemness- and p-EMT-related protein expression was conducted on hTERT^{+}-AM cells subjected to the indicated treatments. **b** Statistical analysis of the relative expression levels of stemness- and p-EMT-related proteins. The data are presented as the means ± SDs. Statistical significance was assessed via two-tailed unpaired Student's t test (**b**).

Figure S13

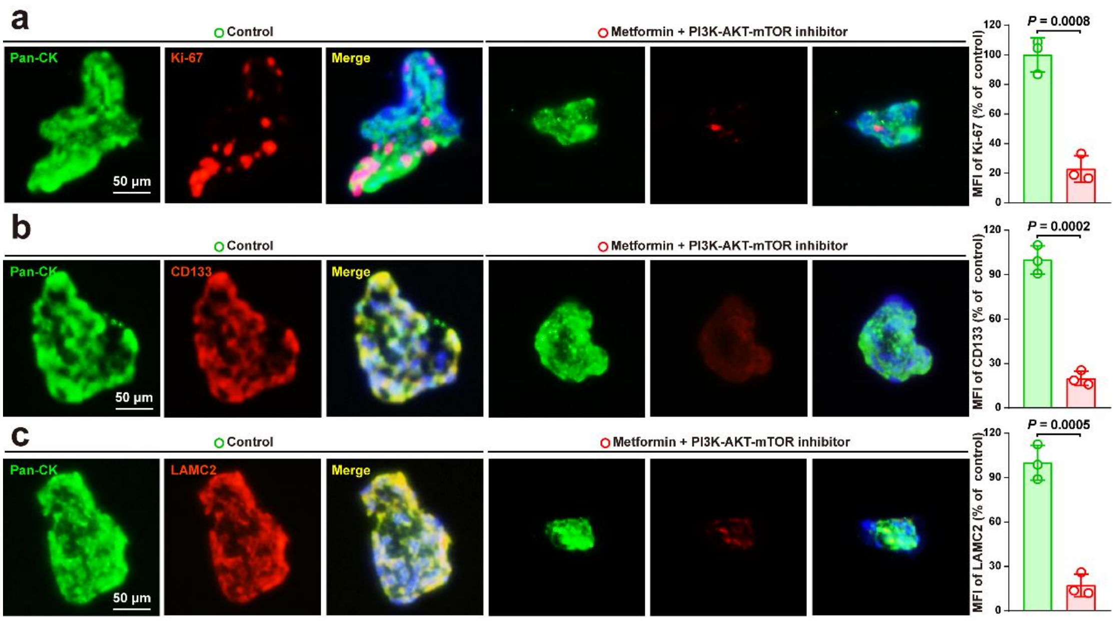

Figure S13. Evaluation of Ki-67, CD133 and LAMC2 expression in ameloblastoma patient-derived organoids (APDOs) treated with or without a PD-L1 inhibitor

a-**c** Immunofluorescence staining assays revealed the expression patterns of Ki-67, CD133, and LAMC2 in APDOs; the scale bar indicates 50 μm (left panel). Quantification of the mean fluorescence intensity for Ki-67, CD133, and LAMC2 expression in APDOs subjected to PD-L1 inhibitor treatment (**a**), PI3K-AKT-mTOR inhibitor treatment (**b**), or combined treatment (**c**) is provided. The data are presented as the means ± SDs. Statistical significance was assessed via a two-tailed unpaired Student's t test for all comparisons (**a**-**c**).

Table S1

Patient No.	Age	Gender	Location	Radiographic appearance	Recurrence	Follow-up (month)
1	45	Male	Mandible	Conventional	No	22
2	55	Male	Mandible	Conventional	No	13
3	66	Female	Mandible	Conventional	Yes	9
4	29	Male	Mandible	Conventional	No	18
5	58	Female	Mandible	Conventional	No	36
6	11	Male	Mandible	Conventional	No	43
7	29	Male	Mandible	Conventional	No	6
8	72	Male	Maxilla	Conventional	Yes	17
9	30	Male	Mandible	Unicystic	No	56
10	17	Male	Mandible	Unicystic	No	31
11	20	Male	Mandible	Unicystic	No	28
12	15	Female	Mandible	Unicystic	No	34
13	12	Female	Mandible	Conventional	No	36
14	56	Male	Mandible	Conventional	No	16
15	38	Male	Mandible	Conventional	No	22
16	11	Female	Mandible	Conventional	No	19
17	13	Male	Mandible	Unicystic	Yes	6
18	26	Male	Mandible	Unicystic	No	12
19	58	Male	Mandible	Conventional	No	18
20	75	Male	Mandible	Conventional	Yes	8
21	65	Male	Mandible	Conventional	No	32
22	52	Female	Mandible	Conventional	No	36
23	46	Male	Mandible	Conventional	No	21
24	30	Female	Mandible	Conventional	No	42
25	10	Male	Mandible	Conventional	No	28
26	26	Male	Mandible	Conventional	No	33
27	42	Male	Mandible	Conventional	Yes	14
28	38	Male	Mandible	Conventional	No	33
29	27	Female	Mandible	Unicystic	No	27
30	39	Female	Mandible	Conventional	Yes	7
31	47	Male	Mandible	Conventional	Yes	6
32	44	Male	Mandible	Conventional	No	42
33	25	Female	Mandible	Conventional	No	28
34	40	Male	Mandible	Conventional	Yes	41
35	72	Male	Mandible	Conventional	Yes	18
36	18	Male	Mandible	Conventional	Yes	24
37	25	Male	Mandible	Conventional	No	27
38	56	Male	Mandible	Conventional	Yes	10
39	24	Male	Mandible	Conventional	Yes	24
40	56	Male	Mandible	Conventional	No	45
41	43	Female	Mandible	Unicystic	No	32

42	57	Female	Mandible	Conventional	Yes	26
43	40	Male	Mandible	Conventional	Yes	7
44	37	Male	Mandible	Conventional	Yes	12
45	33	Female	Mandible	Unicystic	No	34
46	63	Female	Mandible	Conventional	No	36
47	56	Male	Maxilla	Conventional	Yes	18
48	34	Male	Mandible	Conventional	No	25
49	40	Male	Mandible	Conventional	Yes	16
50	36	Female	Mandible	Conventional	No	33
51	19	Male	Mandible	Conventional	Yes	19
52	34	Female	Maxilla	Conventional	No	56
53	22	Male	Mandible	Conventional	No	41
54	65	Male	Mandible	Conventional	Yes	19
55	47	Male	Maxilla	Conventional	Yes	8
56	55	Male	Mandible	Conventional	No	28
57	13	Male	Mandible	Conventional	No	16
58	62	Male	Maxilla	Conventional	Yes	6
59	46	Female	Mandible	Conventional	No	52
60	48	Male	Mandible	Conventional	Yes	8

Table S1. Demographic characteristics of 60 patients with ameloblastoma.

Table S2

Patient No.	Age	Gender	Location	Radiographic appearance
1	33	Male	Maxilla	Multilocular
2	26	Male	Maxilla	Unilocular
3	22	Male	Mandible	Multilocular
4	52	Male	Maxilla	Unilocular
5	24	Male	Mandible	Unilocular
6	35	Female	Both	Multilocular
7	18	Female	Mandible	Unilocular
8	28	Female	Mandible	Unilocular
9	40	Female	Mandible	Multilocular
10	42	Female	Mandible	Unilocular
11	19	Male	Mandible	Multilocular
12	20	Male	Mandible	Unilocular
13	18	Male	Mandible	Unilocular
14	28	Male	Both	Multilocular
15	26	Female	Maxilla	Unilocular
16	43	Male	Mandible	Multilocular
17	18	Male	Mandible	Unilocular
18	22	Male	Maxilla	Multilocular
19	36	Male	Mandible	Unilocular
20	17	Female	Mandible	Unilocular
21	54	Male	Both	Unilocular
22	16	Male	Mandible	Multilocular
23	26	Male	Mandible	Multilocular
24	34	Female	Maxilla	Unilocular
25	32	Male	Mandible	Multilocular
26	18	Female	Mandible	Multilocular
27	43	Male	Maxilla	Unilocular
28	29	Male	Mandible	Unilocular

29	14	Male	Mandible	Multilocular
30	25	Female	Maxilla	Multilocular
31	16	Male	Mandible	Unilocular
32	28	Female	Mandible	Unilocular
33	14	Male	Mandible	Unilocular

Table S2. Demographic characteristics of 33 patients with odontogenic keratocysts.
